# Bidirectional causal relationships between plasma proteins, neuroimaging metrics and risk of Alzheimer's disease

**DOI:** 10.1016/j.tjpad.2026.100619

**Published:** 2026-06-10

**Authors:** Xu Xu, Lintong Li, Wei Huang, Ying Yang, Xu Li, Pei Wang, Mengmeng Zhao, Huiliang Zhang, Chaoming Yuan

**Affiliations:** a Department of Neurology, The Third People's Hospital of Yunnan Province, China; b Department of Neurology, The Second Affiliated Hospital of Dali University, China; c Department of Endocrinology, The Third People's Hospital of Yunnan Province, China; d Department of Endocrinology, The Second Affiliated Hospital of Dali University, China; e Department of Neurology, Yiliu Subdistrict Community Health Service Center of Guandu District, Kunming City, China; f Department of Neurology, Shalatuo Township Health Center, Yuanyang City, China; g Department of Anesthesia, Critical Care and Pain Medicine, Massachusetts General Hospital, Harvard Medical School, Boston, MA 02114, USA; h Department of Neurology, Zhengzhou Emergency Medical Rescue Center, Zhengzhou, China; i Department of Infectious Diseases, Tongji Hospital, Tongji Medical College and State Key Laboratory for Diagnosis and Treatment of Severe Zoonotic Infectious Disease, Huazhong University of Science and Technology, Wuhan 430000, China

**Keywords:** Alzheimer's disease, Neuroimaging metrics, Proteomic, Mendelian randomization

## Abstract

**Background:**

Changes in neuroimaging metrics are among the first detectable pathophysiological alterations in Alzheimer's disease (AD). Proteins are closely linked to fluctuations in neuroimaging metrics. Therefore, the analysis of the proteomic signature associated with neuroimaging metrics holds significant promise for uncovering therapeutic targets that contribute to AD.

**Methods:**

GWAS data concerning the Brain Imaging Data Structure (BIDs). The AD cohort comprised a total of 401,661 individuals diagnosed with AD, alongside 10,520 control participants. For a bidirectional MR analysis involving neuroimaging metrics, proteomics, and AD, the methods utilized included inverse variance weighted (IVW), MR Egger, weighted median, weighted mode, and the Wald ratio approaches.

**Results:**

We identified 12 neuroimaging metrics that demonstrate significant relevance to AD (thickness of the left total hemisphere, volume of the right thalamus, and et al.). These metrics are structural magnetic resonance imaging (MRI) biomarkers that remain stable throughout the entire course of AD, from the preclinical stage through mild cognitive impairment (MCI) to dementia. Additionally, we found a substantial number of 1633 proteins that also show a noteworthy causal relationship with AD. Functional enrichment analysis indicated that these proteins were predominantly focused within various pathways linked to AD, encompassing those involved in the synaptic vesicle cycle, synaptic membranes, neurotransmitter release, and the activity of GABA receptors. In addition, our research indicates that the significant relationships observed between the identified proteins and AD are influenced by neuroimaging metrics. Notably, we found that these neuroimaging metrics play a crucial role in mediating a substantial 67% of the inverse relationship that exists between PTPRC and the phenotypic characteristics associated with AD.

**Conclusions:**

This study successfully establishes a connection between proteomic and neuroimaging metrics, as well as the AD that influence them. By creating this relationship, the research offers important information that aids in comprehending the intricate mechanisms involved in AD.

## Introduction

Alzheimer’s disease (AD), a common neurodegenerative disorder, is recognized as the most prominent form of senile dementia, and achieving efficient clinical remission remains challenging [1]. Clinical symptoms are manifested in progressive decrease in memory, impaired executive function and disability to routine daily activity. However, age of onset, family history, and the presentation of noncognitive symptoms vary with each individual [2]. Ramifications of AD on family caregivers, dementia workforce and the society are influential as the aging of population becomes more pronounced. Although significant progress has been made in the field of therapeutic targets research, the underlying biology of AD is still not fully understood. The well-known hallmarks of AD, including the pathological accumulation of Aβ plaques and Tau neurofibrillary tangles, represent only a segment of the complex pathophysiological mechanisms involved in the disease [3,4]. It is crucial to identify further therapeutic targets, particularly in light of the lack of clinical effectiveness shown by therapies aimed at Aβ and Tau.

Magnetic Resonance Imaging (MRI) remains a crucial tool in examining the individual differences associated with neuroimaging metrics in AD. The use of MRI allows researchers and clinicians to visualize structural changes in the brain, providing important insights into the progression of AD [5,6]. Notably, changes in neuroimaging metrics often represent some of the earliest pathophysiological alterations observable in AD conditions. These modifications can be detected many years (potentially even decades) prior to the emergence of obvious clinical symptoms, highlighting the significance of early diagnosis and monitoring in the management of AD [7,8]. Moreover, longitudinal MRI has the potential to serve as a valuable tool for tracking disease progression at various stages, as well as for forecasting the likelihood of healthy individuals developing brain disorders in the future [9]. Collectively, measures derived from MRI regarding brain structure may be instrumental in enhancing the processes of diagnosing, stratifying, predicting, and monitoring a range of brain disorders.

The presence of proteins in CSF is a well-established indicator of alterations in neuroimaging metrics associated with AD. Notably, specific proteins such as total tau and neurofilament light chain are particularly pertinent to understanding AD [10,11]. Research has demonstrated that the levels of these proteins correlate with the severity of neurodegenerative changes, providing valuable insights into disease progression. Furthermore, studies have shown that a reduction in complement proteins is linked to the degree of brain atrophy observed in patients with mild cognitive impairment. This association underscores the potential of using CSF protein levels as biomarkers in the assessment and monitoring of AD [12,13]. Furthermore, recent studies have highlighted a significant relationship between the expression levels of CSF proteins (Aβ−42, p-tau181) and changes in neuroimaging metrics, particularly in the context of various brain disorders, as well as in the aging process [14,15]. This association suggests that fluctuations in CSF protein profiles may serve as potential biomarkers for understanding the structural alterations that occur in the brain due to neurological conditions and the natural progression of aging. Such findings underscore the importance of further research in this area to elucidate the underlying mechanisms and develop targeted interventions.

Nevertheless, these initial studies exhibit significant shortcomings, often concentrating exclusively on a narrow range of proteins and neuroimaging metrics. Consequently, they do not succeed in offering a thorough overview of the intricate and varied relationships that exist among proteins and neuroimaging metrics. This deficiency in research representation raises important questions about the biological mechanisms regulating these connections. Moreover, it is still uncertain whether the links identified between various proteins and neuroimaging metrics demonstrate unique and identifiable patterns. To delve deeper into the relationships involving proteins, neuroimaging metrics, and AD, MR analyses were conducted, utilizing the genetic data present in the dataset. This methodological strategy sought to identify potential associations that could exist among these factors. The research also investigated the possibility of neuroimaging metrics acting as a mediating variable in the context of proteins and AD. Ultimately, the goal is to create a comprehensive atlas that visualizes the relationships between proteins, neuroimaging metrics, and their implications for AD, thereby deepening our insight into these complex interactions.

## Methods

### Data acquisition

In our comprehensive analysis of the UKB dataset, we successfully identified a total of 2146 unique neuroimaging metrics that were included in the initial dataset release [16]. Certain areas of the brain have likely been evaluated on several occasions using various imaging methods, leading us to contemplate the possibility of redundant neuroimaging measures present in our dataset. To address this issue, we made a careful decision to retain only those that were obtained using the most widely accepted methodologies in neuroimaging. In particular, we concentrated on metrics that were derived from tract-based spatial statistics and probabilistic tractography, as these techniques are regarded as standard practice in the field. This methodical filtering process resulted in the exclusion of 1201 redundant neuroimaging metrics, significantly simplifying and streamlining our dataset. After the initial filtering process, we identified and excluded an extra 358 neuroimaging metrics sourced from cerebellar brain regions. By implementing these comprehensive curation measures, we were able to significantly enhance our dataset. In the end, we selected 587 neuroimaging metrics that we considered appropriate for further analysis, thereby ensuring our study was based on a solid and dependable dataset (Supplementary Table 1) [17]. The AD cohort comprised a total of 401,661 individuals diagnosed with AD, alongside 10,520 control participants, all derived from the FinnGen datasets (Supplementary Table 2).

### Harmonized process of datasets

Then, we implemented a standardized methodology for the management of datasets in our study. This approach included the deliberate exclusion of SNPs that exhibited a MAF of less than 0.01 from the genome-wide association studies. By doing so, we aimed to enhance the quality and reliability of our findings, ensuring that only genetically relevant SNPs were analyzed in relation to the traits under investigation. By employing this criterion, we are able to focus on genetic variants that are more prevalent within the population, thereby improving the validity of our results. Concentrating on these more common variants heightens the possibility that they may significantly contribute to the association results we observe, allowing for a more accurate interpretation of their potential role in the genetic landscape [18].

### Genetic correlation assessment

Before performing MR analyses, we conducted an analysis of genetic correlation to explore the potential connections between neuroimaging metrics and AD. To facilitate this study, we utilized the LDSC approach, specifically crafted to effectively detect and evaluate genetic correlations. Within the framework of our study, we established a significance threshold, setting the p-value cutoff at less than 0.05 [19,20].

### SNP selection

We adopted a statistical threshold of *P* ≤ 5e-8 in conjunction with an r² value of 0.001. This approach allowed us to make informed choices about the SNPs included in the analysis while employing the linkage disequilibrium (LD) clustering algorithm to identify and eliminate SNPs that might pose issues related to potential LD. This step was critical as it helped to alleviate the complexities introduced by linkage imbalance effects that could distort results. Furthermore, to bolster the accuracy and reliability of the MR analysis, we established gene windows for the SNPs at a distance of 100 kilobases. This decision was made to improve the precision of our analysis and to ensure that only the most relevant genetic variants were taken into account [21].

### MR analysis

Then, we implemented a variety of advanced analytical techniques to explore the possible causal connections between brain structure, proteins, and AD. One of the primary methods we utilized was the IVW method. We also employed the MR Egger approach. The MR Egger method is critical in distinguishing genuine causal relationships from those that may be confounded by various extraneous factors. Moreover, we made use of the weighted median method, a robust statistical technique that allows for the estimation of causal effects even in the presence of outliers within the dataset [22]. Lastly, we incorporated the weighted mode method into our analysis, which emphasizes identifying the most prevalent causal effect among the array of instrumental variables generated [23]. This approach provides further insight into the dominant relationships at play among the variables under investigation. By utilizing this diverse set of methodologies, we aimed to conduct a thorough examination of the complex interplay between brain structure, proteins, and AD.

### Quality control

The F-value was computed to assess the extent to which the chosen SNPs explained the variance associated with proteins, brain structure, and AD, along with their related characteristics. This evaluation not only enhanced our SNP selection strategy but also facilitated a more thorough analysis in subsequent stages. Specifically, in instances where a single SNP was pertinent to our study, we employed the Wald ratio method for deeper insights. In cases involving between one and three SNPs, a fixed effects model was used to maintain coherence in our results [24]. Conversely, when scenarios included three or more SNPs, a random effects model was preferred due to the potential for increased variability [25]. Following the MR analysis, we calculated the odds ratio (OR) for each association, accompanied by its respective 95% confidence interval (CI), which established a statistical basis for accurately interpreting the strength and reliability of these associations.

### Enrichment analysis

To further explore the biological implications of the identified proteins, GO and KEGG enrichment analyses were conducted. For these analyses, gene sets were carefully filtered based on specific parameters, including a size range of 5 to 5000 genes and a significance threshold of adj.P.Val less than 0.05.

### Sensitivity analysis

To evaluate the effectiveness and dependability of MR analysis in linking various exposures with their related outcomes, we utilized an array of statistical methods and tests. Among these were the I² statistic for heterogeneity, Cochrane's Q test, Egger's intercept test, the MR-PRESSO method, and a leave-one-out sensitivity analysis [26,27]. A *P*-value below 0.05 is regarded as a sign of considerable heterogeneity among various populations, a situation that the I² statistic quantitatively represents.

## Results

### Instrumental variables (IVs) selection

**Fig. 1:**
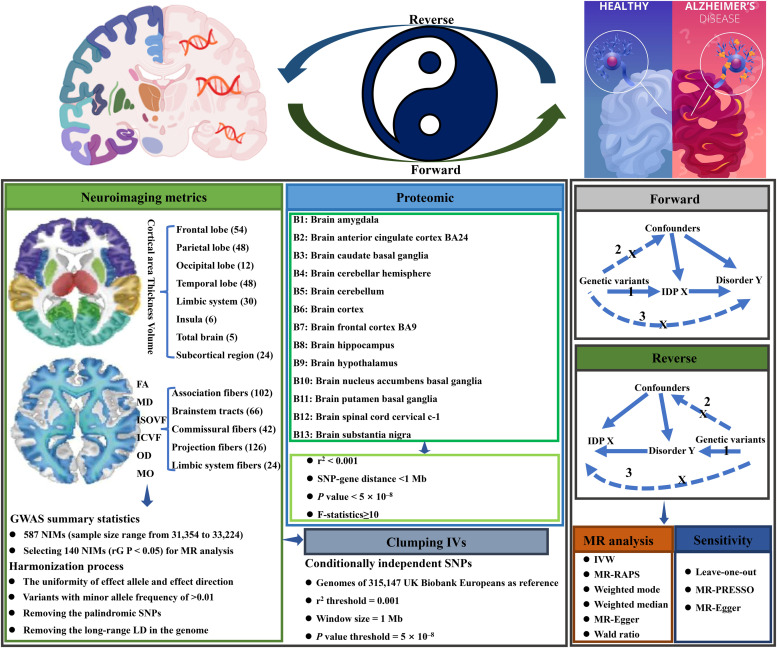
Workflow of this study.

The study design was established and illustrated in Fig. 1. The LDSC analysis provided compelling evidence of a notable genetic correlation, which was established with a P value of less than 0.05 across 65 pairs of neuroimaging metrics-AD (Supplementary Table 3). Following this, we undertook a thorough evaluation to detect the outlier IVs by utilizing RadialMR. This method is specifically designed to assess deviations from expected patterns of IVs, allowing us to critically examine the integrity of our data (Supplementary Tables 4 and 5). To further bolster the credibility of our results, we also took proactive steps to remove certain instrumental variables from our analysis (Supplementary Table 6). These deliberate measures were put in place to enhance the robustness of our study, with the overarching aim of reducing the risk of drawing biased conclusions.

To enhance the reliability of our MR estimates and to clarify the true causal direction between the variables under investigation, we utilized the F-statistic as a key analytical tool. The F-statistic values we obtained for the selected pairs of variables were impressively strong, with every calculated value meeting or surpassing the threshold of 30. The findings reveal that the instrumental variables identified in our analysis explained a significantly larger proportion of variance in the exposure variable as opposed to the outcome variable. This observation not only reinforces the validity of our causal inferences but also highlights the effectiveness of the methodological approaches we utilized in establishing these relationships (Supplementary Table 6).

### Neuroimaging metrics to AD by MR

**Fig. 2:**
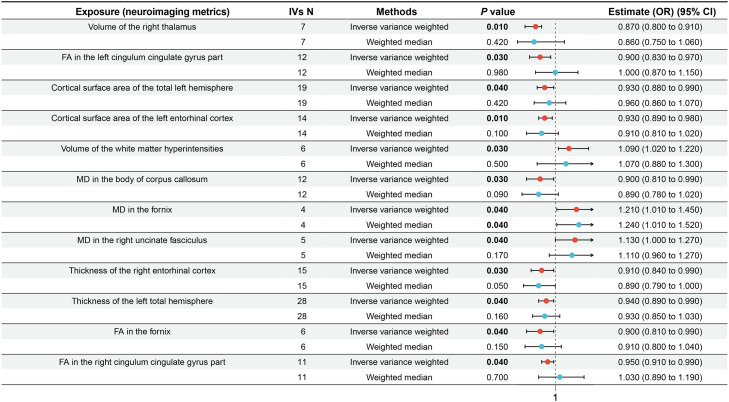
Forward MR to detect the relationship with AD and neuroimaging metrics by using the IVW method.

The forward MR aimed to explore the causal link between alterations in neuroimaging metrics in different regions and the occurrence of AD. This analysis specifically focused on identifying the causal links between neuroimaging metrics and the occurrence of AD, which reveals a total of 12 distinct NIMs that exhibited a significant causal influence on the development of AD (Fig. 2, Supplementary Table 7). Among the notable findings, higher mean diffusivity (MD) in the fornix (OR=1.21, CI 1.01–1.45, *P* = 0.04), in the right uncinate fasciculus (OR=1.13, CI 1.00–1.27, *P* = 0.04), and volume of the white matter hyperintensities (OR=1.09, CI 1.02–1.22, *P* = 0.03) was associated with increase in the likelihood of developing incidentally identified AD. On the contrary, lower of volume of the right thalamus (OR=0.84, CI 0.80–0.91, *P* = 0.01), MD in the body of corpus callosum (OR=0.90, CI 0.81–0.99, *P* = 0.03), thickness of the right entorhinal cortex (OR=0.91, CI 0.84–0.99, *P* = 0.03), fractional anisotropy (FA) in the fornix (OR=0.90, CI 0.91–0.99, *P* = 0.04), cortical surface area of the total left hemisphere (OR=0.93, CI 0.88–0.99, *P* = 0.04), cortical surface area of the left entorhinal cortex (OR=0.93, CI 0.89–0.98, *P* = 0.01), thickness of the left total hemisphere (OR=0.94, CI 0.89–0.99, *P* = 0.04), FA in the right cingulum cingulate gyrus part (OR=0.95, CI 0.91–0.99, *P* = 0.04), and FA in the left cingulum cingulate gyrus part (OR=0.90, CI 0.83–0.97, *P* = 0.03) is linked to a significant increase of risk for incidentally identified AD.

### Proteins associated with neuroimaging metrics

**Fig. 3:**
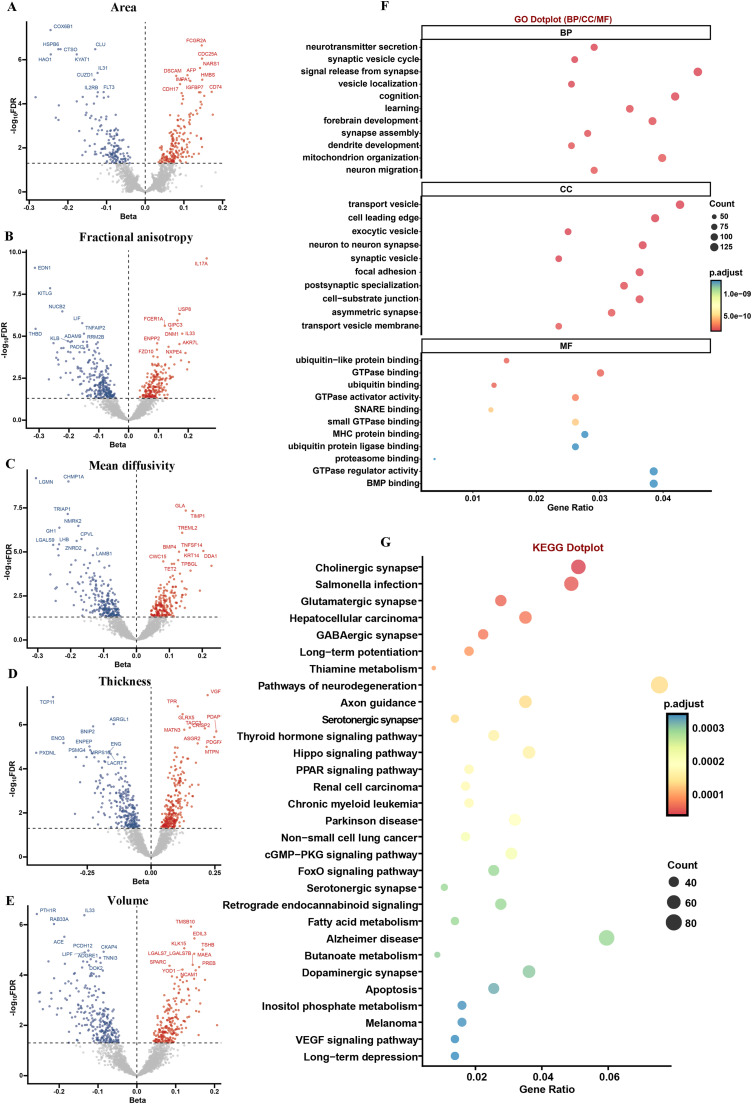
Forward MR to detect the relationship with protein and neuroimaging metrics. Each dot indicates the strongest correlation between the protein and neuroimaging metrics in area (A), fractional anisotropy (B), mean diffusivity (C), thickness (D), and volume (E). (F) GO and (G) KEGG enrichment analysis on the neuroimaging metrics-associated proteins.

Subsequently, researchers identified 2202 significant correlations involving 1633 proteins and various neuroimaging metrics. To validate these results, adjustments for multiple comparisons were made utilizing the FDR method, specifically applying a P_FDR_ threshold of 0.05. Interestingly, the proteins exhibiting the strongest associations differed among the various categories, highlighting a complex interaction between protein expression and particular brain structural measurements (Figs. 3A-2D, Supplementary Table 8). This diversity emphasizes the necessity of accounting for the specific context of each category when examining protein associations with neuroimaging metrics. Functional enrichment analysis indicated that these proteins were predominantly focused within various pathways linked to AD, encompassing those involved in the synaptic vesicle cycle, synaptic membranes, neurotransmitter release, and the activity of GABA receptors (Fig. 3E-F).

### Protein to neuroimaging metrics by MR

**Fig. 4:**
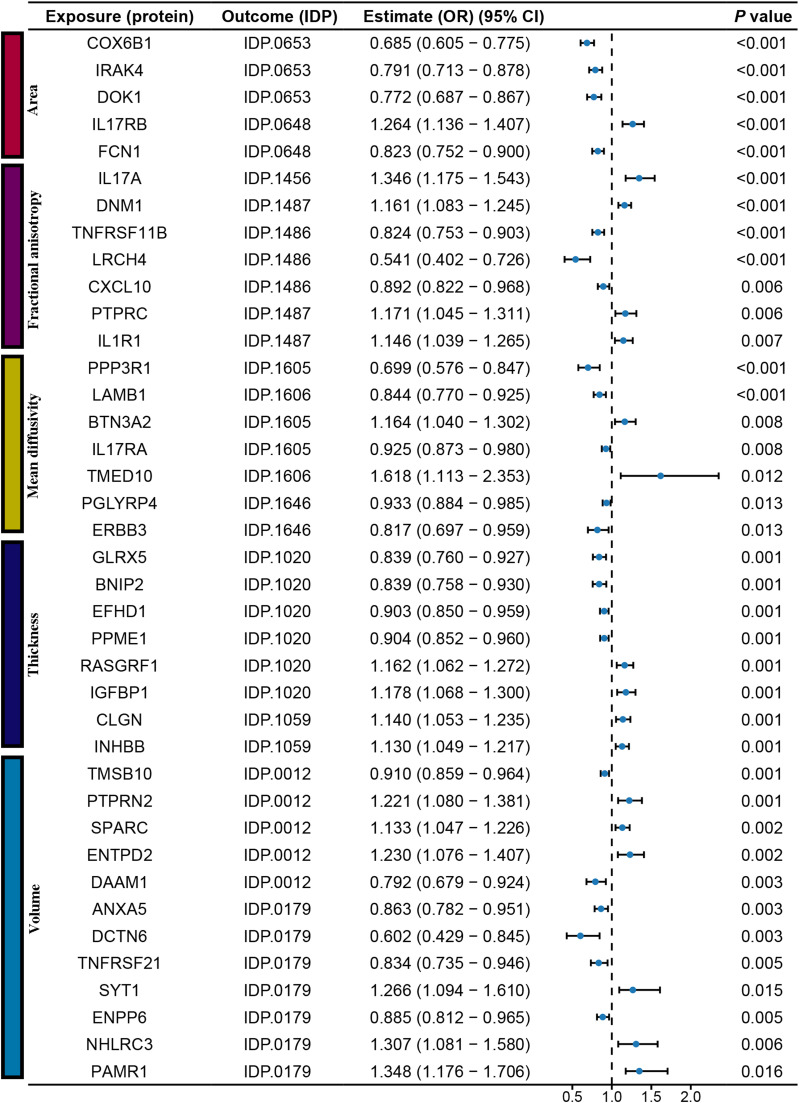
The forest plot shows the significant protein relationships of AD.

In order to enhance our comprehension of the significant connections observed between protein expressions and 12 neuroimaging metrics, we carried out MR analyses. This research concentrated on 39 proteins and 12 neuroimaging metrics that exhibited noteworthy statistical associations (Supplementary Table 9). Notably, an increase of IRAK4 protein expression in the area is linked to a significant increase of 26% in the likelihood of experiencing risk for cortical surface area of the total left hemisphere (OR=1.26, CI 1.14–1.41, *P* < 0.001) (Fig. 4). Subsequently, within the framework of sensitivity analysis, MR analyses showed no signs of directional pleiotropy, as demonstrated by the MR-Egger intercept tests. Furthermore, there was no indication of horizontal pleiotropy, which was evaluated through the MR-PRESSO method. This result implies that the fundamental assumptions of the instrumental variable analysis are valid for the majority of the relationships investigated (Supplementary Table 10).

### Proteins to AD by MR

In order to investigate the potential correlation between 39 proteins associated with the risk of developing AD, we performed MR analyses. Our examination of the links between 39 proteins and AD revealed a total of 10 noteworthy relationships (Supplementary Table 11). These important protein-disease interactions emphasize the crucial role that particular proteins might have in the onset or progression of specific health issues. The results highlight the need for additional exploration of these associations to enhance our comprehension of the disease's underlying mechanisms and possibly lead to improvements in treatment approaches. Then, the outcomes of the sensitivity analysis, which were performed using MR analyses, specifically targeting the relationship between proteins and AD, are comprehensively presented in Supplementary Table 12. Furthermore, regarding the sensitivity analysis, the MR analyses indicated no evidence of directional pleiotropy, a conclusion supported by the MR-Egger intercept tests. Additionally, the assessment for horizontal pleiotropy, conducted using the MR-PRESSO method, yielded no significant indications of this phenomenon. Consequently, this strengthens the legitimacy of our conclusions regarding the intricate interaction between proteins and AD.

### AD to proteins by reverse MR

Then, we successfully discovered 5 proteins significant associations linking AD (Supplementary Table 13). This finding underscores a strong correlation between the presence of AD and the specified proteins, thereby highlighting the importance of this relationship within the broader context of AD research. Furthermore, regarding the sensitivity analysis, the MR analyses indicated no evidence of directional pleiotropy, a conclusion supported by the MR-Egger intercept tests. Additionally, the assessment for horizontal pleiotropy, conducted using the MR-PRESSO method, yielded no significant indications of this phenomenon (Supplementary Table 14).

### Neuroimaging metrics mediated association between proteins and AD

**Fig. 5:**
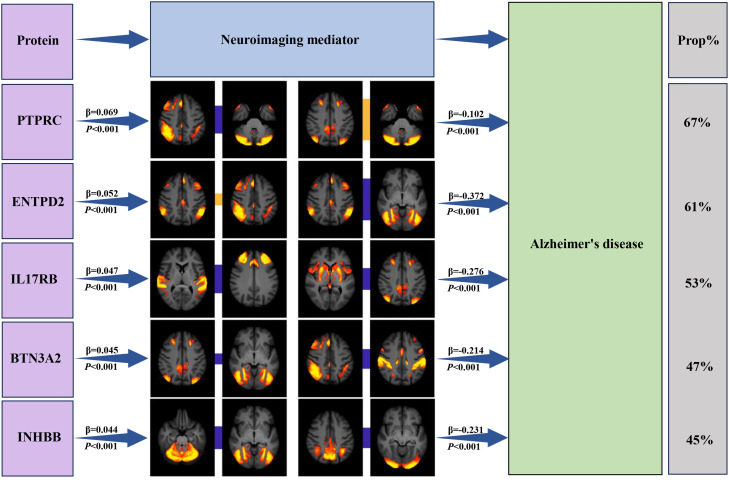
The mediation effect of neuroimaging metrics on association between protein and AD.

Considering the association of the proteins examined with particular neuroimaging metrics, along with the substantial existing literature on the links between neuroimaging metrics and AD, we aimed to investigate another dimension of this topic. Our specific objective was to assess whether neuroimaging metrics could function as a mediator between the protein levels and AD. This examination is vital for unraveling the intricate interactions at play and may provide valuable insights into the potential mechanisms that drive these relationships. The analysis uncovered five significant mediation pathways (*P_FDR_*<0.05). Remarkably, neuroimaging metrics emerged as a complete mediator, explaining 67% of the correlation noted between PTPRC and AD (Fig. 5, Supplementary Table 15). This implies that alterations in these particular brain regions could be essential for comprehending the influence of PTPRC on the onset or advancement of AD.

## Discussion

The research presents a wealth of insights into the proteomic characteristics that are associated with neuroimaging metrics in patients with AD. Among the 587 measures analyzed, 12 were identified as having significant associations with AD. Notably, the proteins that exhibited substantial correlations with various aspects of neuroimaging metrics also indicated links to the risk of developing AD. This finding implies that these proteins are instrumental in influencing both neuroimaging metrics and the risk factors contributing to AD. Furthermore, the study revealed substantial associations between the identified proteins and AD, suggesting that these relationships may be affected by the structural measurements of the brain as they pertain to protein functions. Such connections underscore the complexity and interdependence of protein actions and brain structure in the context of AD. Overall; the research contributes to a sophisticated framework that delineates the intricate patterns of relationships between large-scale proteins and brain architecture. This comprehensive atlas offers valuable insights into the biological foundations of brain health and underscores the potential implications for understanding and addressing AD.

Studies based on observations have indicated a connection between neuroimaging metrics and AD; however, it remains unclear if these relationships are inherently causal. This study successfully identified a set of 12 neuroimaging metrics that demonstrate significant relevance to AD. Studies have shown that lower thickness of the left total hemisphere in AD is primarily attributed to the combined effects of left-sided Tau protein dissemination across brain regions, increased susceptibility of the left-sided highly metabolic association cortex, disruption of the default mode network circuit, and chronic neuroinflammation [28]. These factors trigger progressive loss of neurons and synapses, ultimately leading to widespread thinning of the cortical gray matter [29]. In addition, a significant lower thickness of the left total hemisphere during the aMCI stage can predict progression to AD [30]. Studies have shown that in asymptomatic, Aβ-positive preclinical AD, the MD of the fornix increases first, preceding hippocampal atrophy and cortical thinning, and is one of the earliest white matter markers of damage to the medial temporal lobe memory circuit [31]. The increase in MD in the fornix in AD is mainly mediated by Aβ and tau pathology-induced microstructural damage of white matter, accompanied by disconnection of the limbic memory circuit and secondary trans neuronal degeneration, which ultimately leads to increased water molecule diffusion [32]. Furthermore, the association between increased volume of the white matter hyperintensities and AD is not mediated by direct AD pathology (Aβ and Tau), but by the synergistic interaction between cerebral small vessel disease (CSVD)-induced ischemic damage and AD neurofibrillary degeneration, which accelerates AD progression and exacerbates cognitive impairment [33,34]. By establishing a connection between these metrics and the disease's development, the research opens new avenues for further exploration into how these neuroimaging indicators could be leveraged for early diagnosis and intervention strategies in AD.

The impact of proteins on neuroimaging metrics and their association with AD underscores the essential need to investigate the intricate interactions between these factors in a clinical setting. Gaining insight into how proteins influence neuroimaging metrics and their role in the onset or advancement of AD can yield important information for treatment approaches and interventions. In the investigation of protein associations with neuroimaging metrics, 1633 proteins stood out due to their significant correlations. Functional enrichment analysis indicated that these proteins were predominantly focused within various pathways linked to AD. Such findings underscore the importance of these proteins in understanding the molecular mechanisms underlying brain health and disease. The analysis yielded findings that pinpointed five distinct proteins demonstrating a noteworthy correlation with AD. These proteins include INHBB, IL17RB, PTPRC, BTN3A2, and ENTPD2. Importantly, this association was determined to be influenced by alterations in neuroimaging metrics, suggesting a complex interplay between these proteins and the anatomical changes observed in the context of AD. The β-subunit of the INHBB protein activates activin B, which is closely associated with neurological disorders [35]. BTN3A2 is a notable member of the immunoglobulin superfamily, and it plays a crucial role in the response of T-cells within the framework of the adaptive immune system [36,37]. In recent research, BTN3A2 has been found to have significant associations with various psychiatric and neurological conditions, including AD [36]. Previous studies have provided evidence that genetic variants of BTN3A2 exert pleiotropic effects on AD. Moreover, it has been suggested that heightened expression of BTN3A2 could increase the risk for AD by influencing excitatory synaptic function [38]. ENTPD2 is crucial for the regulation of extracellular ATP levels, which serve as a significant modulator of neuroinflammation and the survival of neurons [39]. In the context of AD, there is an increase in the activity of ENTPD2, which disrupts this essential protective mechanism. As a result, there is an elevation in extracellular ATP levels that is partly fueled by the extensive neuronal death characteristic of AD. This increase in ATP activates P2 × 7 receptors on microglial cells, leading to an excessive activation of these immune cells in the brain. Such hyperactivation of microglia further amplifies neuroinflammation, creating a detrimental feedback loop that contributes to the progressive nature of neurodegeneration in AD [40]. PTPRC is crucial for regulating the immune system, predominantly expressed in brain microglia, which has a significant impact on the pathology of AD [41]. This protein is instrumental in modulating the responses associated with neuroinflammation by regulating the activation of microglia, which consequently leads to the release of pro-inflammatory cytokines [42]. The increase in neuroinflammation has been linked to the progression of AD, highlighting the importance of PTPRC in this context. Moreover, the function of PTPRC in the regulation of microglial activity is critical for the effective management of brain inflammation and the advancement of AD [43]. The interaction of PTPRC with various proteins, such as GFAP and ITGB2, is essential for maintaining cellular dynamics and immune functions within the brain in the context of AD [44]. These interactions are crucial for ensuring the proper functioning of microglia and the overall immune response in the brain, further underscoring the significance of PTPRC in the progression of neurodegenerative diseases.

Considering the findings of this study, it is crucial to recognize various possible limitations. The predominance of European ancestry within the UK Biobank presents a significant limitation to the cross-ethnic generalizability of findings. This restricts the applicability of research outcomes across diverse populations, thereby potentially skewing our understanding of health disparities and genetic influences in various ethnic groups. Moreover, the current imaging metrics utilized in assessing pathological subtypes are insufficiently refined. This inadequacy may lead to unmeasured confounding variables that complicate the interpretation of imaging results, thus hindering precise diagnosis and treatment approaches. Furthermore, the absence of robust methodologies such as qPCR, immunohistochemistry, or validation at the protein level using local clinical tissue samples raises concerns about the reliability of the findings.

In conclusion, this study capitalizes on the remarkable opportunity offered by vast data drawn from genetic, proteomic, and neuroimaging investigations, offering a comprehensive map of proteins associated with neuroimaging measures. The proteins identified are associated with AD conditions, underscoring their significance as pivotal biomarkers for the evaluation of AD. Moreover, it was found that neuroimaging measures play a mediating role in the relationship between plasma proteins and overall brain health. By uncovering these relationships, this research enhances the understanding of the connections among plasma protein markers, neuroimaging metrics, and AD, paving the way for the exploration of the mechanisms that underlie brain disorders and for identifying potential therapeutic options.

## Data statement

GWAS statistics of brain neuroimaging metrics were collected from BIG40 web browser (https://open.win.ox.ac.uk/ukbiobank/big40).

## Fundings

This work was received The Third People's Hospital of Yunnan Province Intramural Research Project (2022SSYKT005), 2026 Yunnan Provincial Department of Education Scientific Research Fund Projects (2026J0904).

## Declaration of the use of generative AI and AI-assisted technologies in scientific writing and in figures, images and artwork

I confirm that I have not used any AI at all.

## Ethical statement

Not applicable.

## Studies on human and/or animal statement

Not applicable.

## Informed consent

Not applicable.

## Approval ± registration number

Not applicable.

## CRediT authorship contribution statement

**Xu Xu:** Project administration, Methodology, Formal analysis, Data curation. **Lintong Li:** Validation, Methodology, Investigation, Formal analysis, Data curation. **Wei Huang:** Validation, Methodology, Formal analysis. **Ying Yang:** Software, Formal analysis, Data curation. **Xu Li:** Methodology, Formal analysis, Data curation. **Pei Wang:** Validation, Formal analysis, Data curation. **Mengmeng Zhao:** Validation, Supervision, Software. **Huiliang Zhang:** Formal analysis, Data curation. **Chaoming Yuan:** Writing – review & editing, Writing – original draft, Project administration, Funding acquisition.

## Declaration of competing interest

The authors report no conflicts of interest in this work.

## Appendix Supplementary materials

Supplementary Table 1: GWAS Summary-level data of neuroimaging metrics. Image, application 1

Supplementary Table 2: GWAS summary-level data of AD. Image, application 2

Supplementary Table 3: The results of genetic correlation between neuroimaging metrics and AD. Image, application 3

Supplementary Table 4: IVs associated with confounders in forward MR analyses. Image, application 4

Supplementary Table 5: Outliers detected by RadialMR in forward MR analyses. Image, application 5

Supplementary Table 6: Information of IVs for all exposure-outcome pairs in forward MR analyses. Image, application 6

Supplementary Table 7: Forward MR analysis results. Image, application 7

Supplementary Table 8: Association between plasma proteins and brain structures. Image, application 8

Supplementary Table 9: Forward MR analysis results (protein to neuroimaging metrics). Image, application 9

Supplementary Table 10: Pleiotropy assessment for the significant results of forward MR analyses (protein to neuroimaging metrics). Image, application 10

Supplementary Table 11: Forward MR analysis results (protein to disease). Image, application 11

Supplementary Table 12: Pleiotropy assessment for the significant results of forward MR analyses (protein to disease). Image, application 12

Supplementary Table 13: Reverse MR analysis results (disease to protein). Image, application 13

Supplementary Table 14: Pleiotropy assessment for the significant results of reverse MR analyses (disease to protein). Image, application 14

Supplementary Table 15. Statistics for Mediation analysis. Image, application 15
